# A simplified methodology to produce Monte Carlo dose distributions in proton therapy

**DOI:** 10.1120/jacmp.v15i4.4413

**Published:** 2014-07-08

**Authors:** Chris Beltran, Yingcui Jia, Roelf Slopsema, Daniel Yeung, Zuofeng Li

**Affiliations:** ^1^ Department of Radiation Oncology Mayo Clinic Rochester MN; ^2^ St. Jude Children's Research Hospital Memphis TN; ^3^ University of Florida Proton Therapy Institute Jacksonville FL USA

**Keywords:** Monte Carlo, proton therapy, dose calculation

## Abstract

The purpose of this study was to develop a simplified methodology that will produce Monte Carlo (MC) dose distribution for proton therapy which can be used as a clinical aid in determining the adequacy of proton plans produced from the treatment planning system (TPS). The Geant4 Monte Carlo toolkit was used for all simulations. The geometry of the double scatter nozzle in the simulation was a simplification of the treatment nozzle. The proton source was modeled as discrete energy layers, each with a unique energy distribution and weighting factor. The simplified MC system was designed to give the same dose distribution as the measured data used to commission the TPS. After the simplified MC system was finalized, a series of verification comparisons were made between it, measurements, and the clinically used TPS. Comparisons included the lateral profile of a stair‐shaped compensator that simulated a sharp lateral heterogeneity and depth‐dose measurements through heterogeneous materials. The simplified MC system matched measurements to within 2% or 2 mm for all commissioning data under investigation; moreover, the distal edge and lateral penumbra was within 1 mm of the measurements. The simplified MC system was able to better reproduce the measured profiles for a stair‐shaped compensator than the TPS. Both MC and TPS matched the measured depth dose through heterogeneous materials to within 2% or 2 mm. The simplified MC system was straightforward to implement, and produced accurate results when compared to measurements. Therefore, it holds promise as a clinically useful methodology to validate the relative dose distribution of patient treatment plans produced by the treatment planning systems.

PACS number: 87.55.K‐, 87.55.ne

## I. INTRODUCTION

Proton therapy is rapidly becoming accepted as the preferred radiation modality for select tumor sites, particularly pediatric brain tumors.^1^, ^2^, ^3^ Because of the physics of the proton particle, one is able to deliver the desired radiation dose to the target, while at the same time drastically reducing or eliminating radiation dose to surrounding normal tissue, particularly distal to the target. However, these same attributes make accurate calculation of dose deposition in heterogeneous areas very important and prone to errors for the analytical methods used in treatment planning systems (TPS). Unlike photons, where a slight calculation or position error of a few millimeters may result in a slight change in dose, a slight error with protons can result in large dose errors. Current treatment planning systems and quality assurance methods cannot fully eliminate these errors; however, a robust Monte Carlo simulation of the treatment has the potential to enhance the accuracy of proton planning and delivery.

The accuracy of commercial proton TPS in highly heterogeneous areas is a concern. Due to different particle paths, the depth‐dose distribution along a beam line through heterogeneities can be significantly different than through water only, thereby widening the distal falloff. If this effect is not adequately accounted for in the TPS, unwanted dose‐to‐distal normal tissue or underdosing of the target volume may occur.^4^, ^5^ In addition, the lateral scatter of the protons is affected by heterogeneities, thereby making accurate dose calculations difficult.^6^, ^7^

Some investigators have developed customized Monte Carlo dose calculation systems to increase the confidence in treatment delivery and machine performance, while others have developed simplified calculation systems based on Monte Carlo data.^4^, ^8^, ^9^, ^10^, ^11^ These systems are of clinical use only to their institutions and are very time consuming to implement. Due to these limitations and the possible clinical impact on our patients being treated with proton therapy, we undertook this project.

The system described in this manuscript has been used to compare the dose distribution from a commercially available treatment planning system to Monte Carlo calculations.^(12)^ All the patient‐specific items, such as apertures and compensators, are described in that paper. The goal of this manuscript is to describe the methodology used in developing and commissioning the simple and clinically usable Monte Carlo‐based system. It is important that, for each new type of nozzle design, a full Monte Carlo simulation is conducted, including all components of the nozzle in a dynamic 4D setting. This allows absolute calibration and verification of a complex system to be simulated for a variety of cases, measurements of which would be impractical. Unfortunately, this modeling requires intensive computational power, expertise of a Monte Carlo package, and several months or more of detailed geometry coding which may not always be practical in a clinical setting. However, as we will show in this manuscript, one can accurately use Monte Carlo to reproduce the relative dose from the proton therapy unit with a simplified geometry, and use most of the computational time for calculation in the complex target environment (i.e., the patient).

## II. MATERIALS AND METHODS

The Monte Carlo system described herein is based on simplified and time‐independent nozzle geometry and a complex, but time‐independent, source description. This simplification allows the vast majority of the MC calculation time to be spent within the patient geometry and not in the nozzle. Also, detailed time‐dependent geometries are not required, thereby decreasing the overall time needed to create the Monte Carlo system. This is different from other Monte Carlo systems for double scattering proton therapy, where all complex and time‐dependent components are explicitly modeled

A double scatter proton that can cover any range and SOBP up to 20 cm was simulated in this study. All simulations were performed using the Geant4^(14)^ Monte Carlo toolkit (version 9.3). The goal was to match the MC simulation to the measurements used to commission the TPS. The measurements of particular interest were the multiple percent depth‐dose (PDD) measurements and the profile measurements at different depths. At the different depths, the MC system was compared to measurements for at least two PDDs, one with a short spread out Bragg Peak (SOBP) and the other with a large SOBP, and at least three profiles, each at a different portion of the SOBP. A total of 15 SOBPs and 45 profiles were used to commission this simplified MC system. Special attention was paid to the distal edge (80% to 20%) of the SOBPs and the lateral penumbras (80% to 20%) of the profiles. The actual components of the nozzle were not simulated but, instead, the complexity was included in the source description. The details are given in the following sections.

### A. Commissioning the simplified Monte Carlo system

Note: For all commissioning measurement data, depth‐dose curves were taken with a PTW Advanced Markus parallel plate ionization chamber (PTW, Freiburg, Germany) and profile measurements with a Scanditronix EDP 2 electron diode (IBA Dosimetry GmbH, Schuarzenbruck, Germany); all were made in an IBA Blue Phantom scanning water phantom (IBA Dosimetry GmbH).

#### A.1 Geometry description

**Figure 1:**
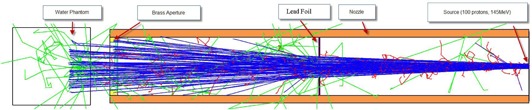
Schematic of the geometry used for the Monte Carlo simulations. Note that the only item in the nozzle is the lead foil. Blue=positively charged particles, red=negative, green=neutral.

Since geometric simplicity was a major concern, the nozzle (container of the scatterers, range modulator wheels, ion chambers, etc.) and the different size snouts (container of the patient‐specific aperture and compensator) were all simplified to a metallic cylinder of inner radius 13 cm and wall thickness of 6 cm. A simple lead foil was placed in the center of the simplified nozzle and was needed because no actual scatterers, range modulators, or ion chambers were placed in the simulated nozzle. The thickness of the lead foil was a parameter to be fit by comparing the simulated depth‐dose curve to the commissioning measurements. In an effort to keep the nozzle simulation simple, lead was chosen as a surrogate for the various high‐Z materials in the nozzle. The commissioning data aperture for the PDDs was a circle with radius of either 5.0 cm or 12.5 cm, and for the profiles, it was a 10 cm square. No compensators were used for these measurements. For the simulation, the proton source was positioned at the top of the nozzle, 230 cm from isocenter, which is the approximate source to isocenter distance of the actual system. Figure 1 is a schematic of the geometry setup.

For comparison to measurements, a large block of liquid water $\left(40\times 40\times 40\;{\text{cm}}^{3}\right)$ was used in the Monte Carlo simulation to represent the scanning water tank $\left(48\times 48\times 41\;{\text{cm}}^{3}\right)$ used to gather the commissioning data. The relative dose was calculated using the mesh tool in Geant4. For commissioning purposes, the mesh size was set to 1 mm^3^ and covered the entire water phantom.

#### A.2 Source description

The description of the source is complex when compared to the geometry. There were three major components to the source: 1) the discrete energy layers; 2) the weighting factor of each layer; and 3) the energy spread of each layer. The details of the beam transport though the actual nozzle have been discussed elsewhere;^(13)^ therefore, we will only give a brief overview of what gives rise to these three components of the proton source.

For the double scattering system, given the penetration depth and the SOBP width, all major machine parameters are fixed and can be found via a spreadsheet provided by the vendor. This spreadsheet contains the conversion algorithm that is inside the therapy control system and also within the treatment planning system. The major parameters are: (i) the proton energy into the nozzle, (ii) combination of primary scatterers, (ii) range modulator, (iv) secondary scatterer, and (v) current modulation.

The discrete energy layers used for our source were derived from five main components: 1) the proton energy into the nozzle; 2) the water‐equivalent thickness (WET) of the primary and secondary scatterers in use; 3) the WET of other nozzle components in the beam line (i.e., ion chambers); 4) the WET of each discrete step in the range modulator; and 5) the weighting factor of each energy layer. Component 4) leads to distinct energy layers. The weighting factor of each energy layer was derived from the current modulation data, the length of each step of the given range modulator, and the start angle of each range modulator. The start angle of the range modulator wheel is measured during commissioning and essentially shortens the length of the first step. The length of the first step (i.e., start angle) was varied around the point found at commissioning to find the best match of the measured data. The WET of the lead foil was taken into account when calculating the energy layers.

Since there were no physical components in the simulated nozzle, except for a lead foil, the energy spread must be calculated. The energy spread used in this work contains two components which were added in quadrature. The first was the spread of the proton beam as it enters the nozzle (i.e., before it interacts with any component within the nozzle). This spread has been determined and is given by Equation 1 in the paper by Paganetti et al.^(4)^ The other component was the range straggling, and is given by Equation 31 in the paper by Ulmer.^(15)^ The range straggling is needed because there are no physical components in the simulated nozzle; therefore, the range straggling that would be present in the actual nozzle due to the physical nozzle components must be explicitly included in the energy spread.

The shape of the proton source was modeled as a circular disk and placed at the upper end of the nozzle, which is 230 cm from isocenter. The size of the disk was varied to give the best match for penumbra width to the measurement data. The angular spread of the beam was set to ensure full and even proton spread at the nozzle exit.

#### A.3 Aperture and compensator

To test the accuracy with an arbitrary aperture and compensator, the MC system and TPS were compared to commissioning measurements that consisted of a circular aperture of radius 6.5 cm and a compensator consisting of three parallel Lucite steps with sharp edges, each 6 cm wide and 2.7, 0.2, and 5.2 cm thick (stair compensator). This created a large heterogeneity parallel to the beam direction. Since the exact dimensions of the aperture and compensator were known, these were accurately simulated.

### B. Comparison between MC, TPS, and measurements

To verify the accuracy and robust nature of the MC system, a series of relative dose distribution comparisons in a solid water phantom were performed. The first set of verification tests were based on comparisons between the MC system, measurements, and the clinically used Varian Eclipse version 8.1 (Varian Medical Systems, Palo Alto, CA) TPS. The verification measurement comparisons were made at several depths in a solid water phantom with the MapCHECK (Sun Nuclear, The Hague, The Netherlands) 2D diode array, which has a 7.07 mm detector spacing in the center $10\times 10\;{\text{cm}}^{2}$ area. This allowed for both relative depth dose and profile measurements. The validity of using MapCHECK for proton measurements has been previously reported to have mm accuracy for relative dose measurements,^(16)^ and verified before use in this study. The phantom consisted of solid water with an artificial bone and lung slab placed in the middle of the phantom. The aperture was a 5 cm by 7 cm rectangle and there was no compensator. The exact geometry and material composition of this phantom were simulated in the MC system. A treatment plan based on the CT of this phantom was created in the TPS. The mesh size in the MC simulation was set to $2\times 2\times 2\;{\text{mm}}^{3}$. The calculation grid size for the TPS was 2.5 mm. To conduct the measurements, several runs were performed; for each run the position of the MapCHECK was lowered in 5 mm increments and additional solid water was added. This kept the heterogeneity at the same place relative to the proton beam and moved the measurement point. To increase the lateral resolution, all measurements were rerun with the MapCHECK shifted 0.25 cm relative to the phantom.

Since measured data is limited in quantity, other verification tests consisted of comparisons between the MC and TPS in a simple water phantom for a wide variety of simple aperture and compensator shapes, each with a variety of proton ranges and modulation widths. The position of isocenter was the center of each SOPB, resulting in a different source‐to‐surface distance for each verification field. Measurements were not required for this comparison because the TPS can accurately calculate the relative dose distribution in a simple water phantom.

## III. RESULTS

### A. Commissioning

**Figure 2:**
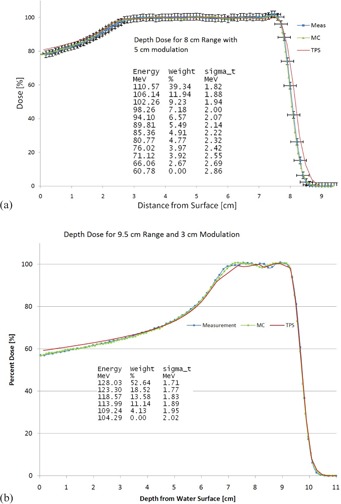
A depth‐dose comparison between the measurements, TPS, and MC is displayed. Also shown is the source description used in the MC simulation. The source description consists of numerous energy layers, with each layer being described by its energy, the weight, and the energy spread (sigma_t) of that layer. Typically, the TPS had a better match to measurement in the distal edge than is shown in (a). The error bars on the measurement shown on (a) are ±2% in dose and ±1.6 mm in distance.

The MC system matched measurements for all commissioning data under investigation to within 2% or 2 mm; moreover, the distal edge and lateral penumbra matched to within 1 mm for all measurements (15 SOBPs and 45 profiles). Figure 2(a) demonstrates the match between measurements, TPS, and MC for a large SOPB. Note the very good agreement (<1 mm) between MC and measurement at the distal edge. Also displayed in that figure is the source description used in that MC simulation. Typically, the TPS matched the measurement at the distal edge better than depicted in this figure. This particular TPS match is an outlier, but is included to show that the MC dose match well. Figure 2(b) contains the same information, except it is for a different depth and small SOPB.

The thickness of the lead foil varied from 0.75 to 6.0 mm. The thickness of the foil was related to, but not directly proportional to, the thickness of the various scatterers used for a given range.

**Figure 3:**
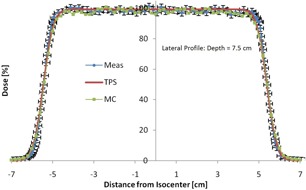
Profile dose comparison between the measurements, TPS, and Monte Carlo are shown. The range was 8 cm and modulation was 5 cm. The error bars on the measurement are ±2% in dose and ±1 mm in distance.

The source position in the MC was fixed at 230 cm from isocenter. The source used can be described as a circular disk of diameter 2.7 cm and an angular spread of 3.44°. Though the source size was a parameter to be fit for each depth, it was found that it and the angular spread could be kept constant. It is important to note that the “apparent” source size does change to the different thickness of the lead foil (i.e., due to the scattering effect of lead, the thicker the lead foil, the larger the apparent source size). Figure 3 shows a profile comparison at depth. Note the very good agreement (<1 mm) between MC and measurement in the penumbra region. All profile commissioning comparisons had similar results to Fig. 3.

**Figure 4:**
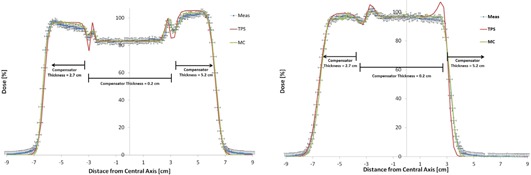
Dose profile comparison of a stair‐shaped compensator in water between measurement, treatment planning system, and the Monte Carlo system at 3.7 cm (left) and 11.7 cm (right) depth. The range was 15.2 cm and modulation was 10.0 cm. The thickness of the compensator is indicated in the plot. The error bars are ±2% in dose and ±1 mm in distance.

The stair compensator has two sharp density heterogeneities at ±3 cm from the central axis. This causes a problem for the TPS as it is not able to properly account for the change in lateral scatter and, therefore, does not reproduce the measured results in this area properly. The MC system was able to reproduce the measured results in the area of the heterogeneity more accurately than the TPS. Figure 4 depicts the stair compensator dose profiles at a depth of 3.7 and 11.7 cm for the TPS, MC, and measurement. Note: 11.7 cm depth is 2.3 cm beyond the Bragg Peak for the 5.2 cm thick side of the stair compensator. Profiles at different depths had similar results. The MC and TPS matched (2% or 2 mm) the depth dose measurements of the stair compensator both on and off central axis.

### B. Verification

**Figure 5:**
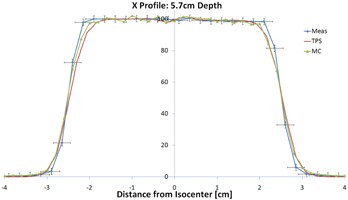
Profiles perpendicular to bone/lung interface in the heterogeneous phantom for the MapCHECK‐based measurement, treatment planning system, and Monte Carlo system are shown. Error bars are ±2% in dose and ±2 mm in distance.

**Figure 6:**
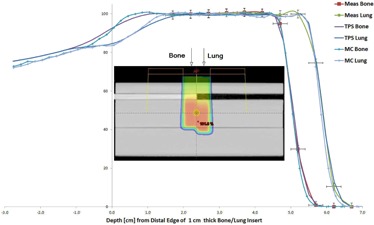
Relative depth dose values for the MapCHECK‐based measurement, treatment planning system, and the Monte Carlo system through both the bone and lung inserts. Error bars on the measured data are ±2% in dose and ±2 mm in distance. The insert image demonstrates the phantom setup with the dose distribution from the TPS.

The comparison between the MapCHECK measurement, TPS, and MC for the heterogeneous phantom had encouraging results. The profiles from the TPS and MC that were parallel but distant to the bone/lung interface at different depths matched the measurements to within the measurement uncertainty (2% or 2 mm). As shown in Fig. 5, profiles perpendicular to the interface showed slight discrepancies between the measurements, TPS, and MC, but were still within the measurement uncertainty. The distal edge of the depth dose through the bone and lung also matched measurements, as shown in Fig. 6. Unfortunately, we do not have measurement data within the heterogeneity itself.

The verification test between TPS and MC consisted of nine different aperture and compensator combinations: three apertures that were 5×7 cm,10×10 cm, and 25 cm in diameter, and three step‐shaped compensators with a 2, 4, and 6 cm variation in the minimum and maximum thickness, giving 54 different SOBPs and 162 profiles (three depths per SOBP). The two systems matched each other to within 2% or 2 mm, except for the profiles of the compensator which had a large variation similar to the stair compensator. For those profiles, the results were similar to Fig. 4.

## IV. DISCUSSION

We have shown that the Monte Carlo system we developed produces accurate results in a water phantom. This implies that the phase space exiting the nozzle is realistic and, therefore, the MC calculation will give a more accurate result than the TPS. We have also shown that, for the explicit purpose of relative dose calculation, the full details of the nozzle are not required. In addition, as the MGH group has shown, when a careful and detailed simulation of the nozzle is performed, modifications to the input current are required in order to match measurements, due to the various uncertainties inherent in simulations of complex systems.^(17)^ Since we are interested in the relative dose calculation within the patient, having an accurate simulation of the phase space exiting the nozzle, which then passes through the aperture and compensator and into the patient, is necessary and sufficient for accurate Monte Carlo calculations within the patient.

These accurate calculations are required when large heterogeneities are present, similar to Fig. 4. This is crucial, as many targets and critical structures in the patient are near heterogeneities, such as bone/air interfaces in sinus cavities and auditory canals. Because of this shortcoming of the TPS, it is possible for an underdose of the target and/or an overdose of critical structures to occur. Routine clinical use of a MC system may help prevent such errors. A manuscript detailing the patient specific procedures and quantifying the difference between this MC system and the TPS for a large cohort of patients has been published.^(12)^

To get a 2% statistical uncertainty in the target area, it takes our system approximately 25 to 30 hours to calculate the phase space that enters the patient, then approximately 5 hours to calculate the dose within the patient CT (which is highly dependent on field size and modulation) on a 2.5 GHz CPU. As stated by Paganetti,^(18)^ with a similar CPU, for their full 4D simulation with the same 2% uncertainty and similar CPU, 4.5 hours are spent on the dose calculation within the CT, but approximately 60 hours to develop the phase space. That is a factor of two reduction in time spent calculating the phase space at the exit of the nozzle with our simplified system. With prior information about the size of the aperture, our system does have the capability to decrease the time required to do the phase space calculation by directing the initial protons more efficiently. For a relatively small field (less than 10 cm by 10 cm), the time can be reduced by approximately a factor of two by eliminating the tracking of wide scattered protons in the nozzle. However, this efficiency is not currently implemented in our system.

## V. CONCLUSIONS

The simplified MC system was straightforward to implement and produced accurate results when compared to measurements. Therefore, it holds promise as a clinically useful methodology to validate the relative dose distribution of patient treatment plans produced by the treatment planning systems.

## Supporting information

Supplementary Material

Supplementary Material

Supplementary Material
