# Supplementary figures and images for: A simplified methodology to produce Monte Carlo dose distributions in proton therapy
Source: J Appl Clin Med Phys. 2014 Jul 8;15(4):2–10. doi: 10.1120/jacmp.v15i4.4413 (PMC5875513; doi:10.1120/jacmp.v15i4.4413)

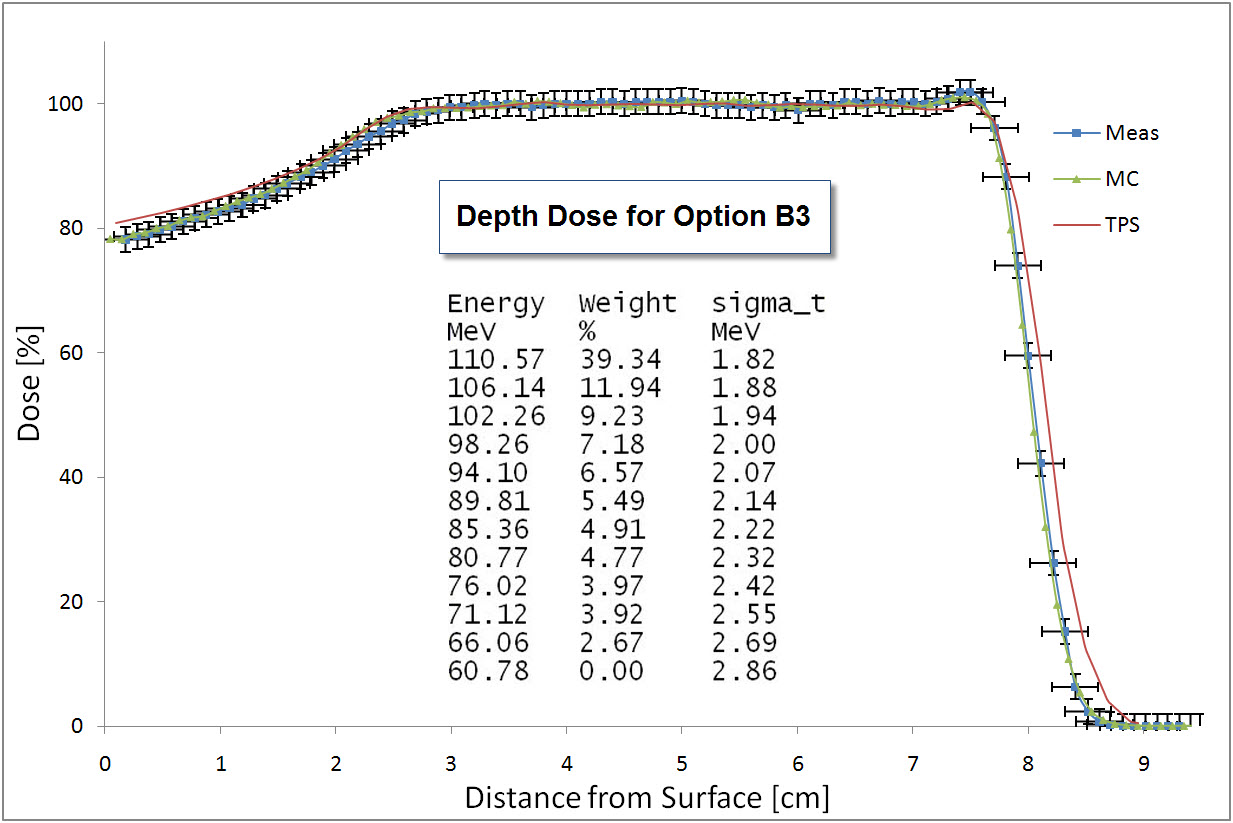

Supplement: Supplementary file 1 — Supplementary Material [file ACM2-15-2-s001.jpg]

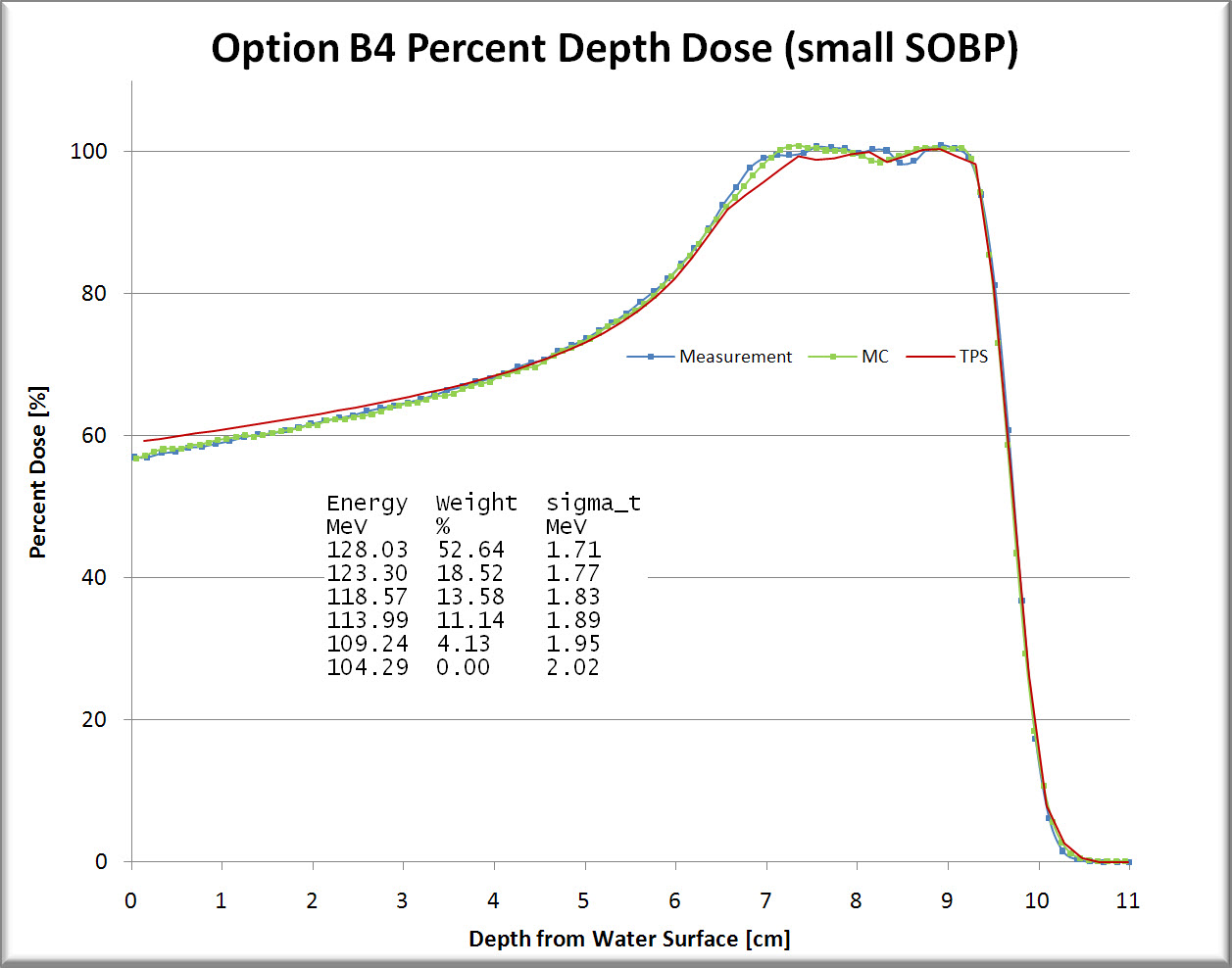

Supplement: Supplementary file 2 — Supplementary Material [file ACM2-15-2-s002.jpg]

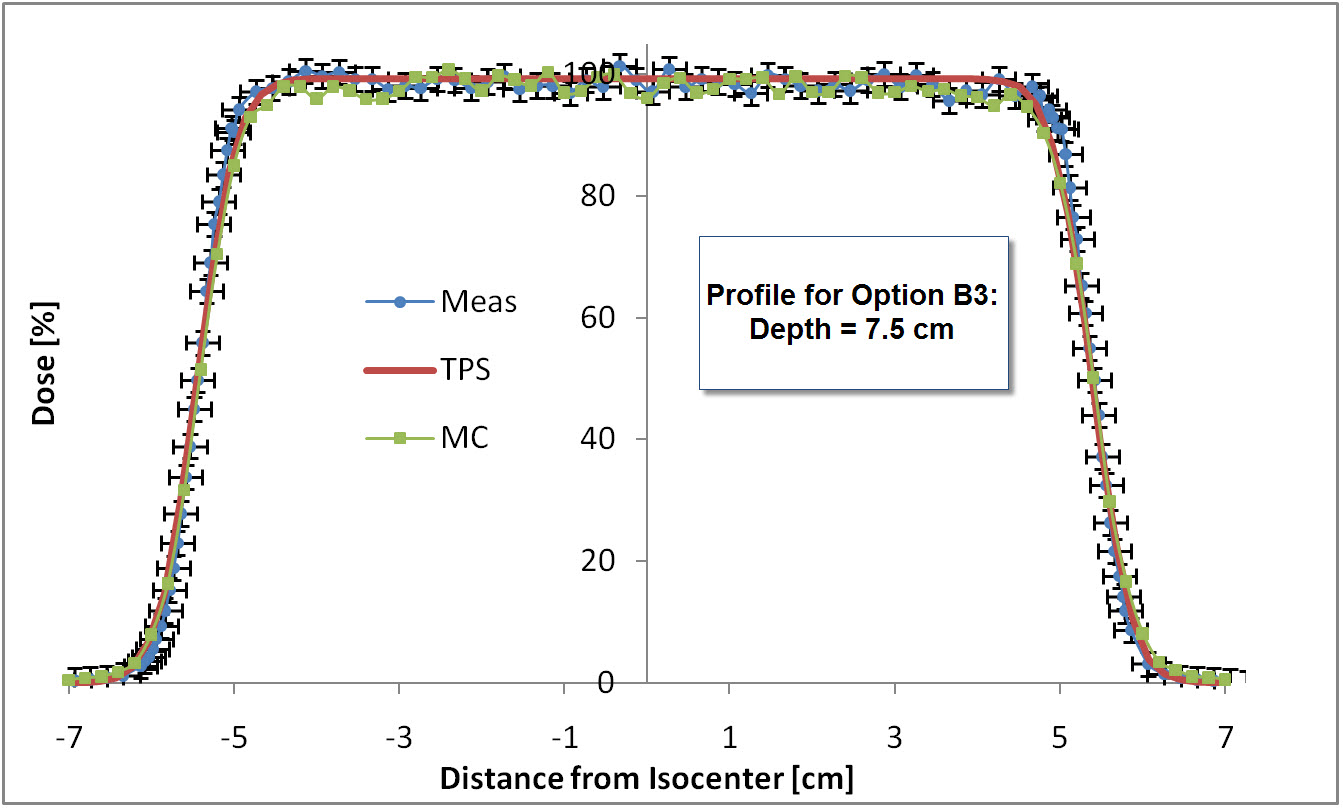

Supplement: Supplementary file 3 — Supplementary Material [file ACM2-15-2-s003.jpg]
